# Clinical efficacy of potassium canreonate-canrenone in sinus rhythm restoration among patients with atrial fibrillation - a protocol of a pilot, randomized, double -blind, placebo-controlled study (CANREN-AF trial)

**DOI:** 10.1186/s13063-020-04277-3

**Published:** 2020-05-12

**Authors:** Rafał Dąbrowski, Paweł Syska, Justyna Mączyńska, Michał Farkowski, Stefan Sawicki, Agata Kubaszek-Kornatowska, Piotr Michałek, Ilona Kowalik, Hanna Szwed, Tomasz Hryniewiecki

**Affiliations:** National Institute of Cardiology, ul. Alpejska 42, Warsaw, 04-628 Poland

**Keywords:** Atrial fibrillation, Cardioversion, Canrenone

## Abstract

**Background:**

Atrial fibrillation (AF) is the most frequent cardiac arrhythmia which increases the risk of thromboembolic complications and impairs quality of life. An important part of a therapeutic approach for AF is sinus rhythm restoration. Antiarrhythmic agents used in pharmacological cardioversion have limited efficacy and potential risk of proarrhythmia. Simultaneously, underlying conditions of AF should be treated (e.g. electrolyte imbalance, increased blood pressure, neurohormonal disturbances, atrial volume overload). There is still the need for an effective and safe approach to increase AF cardioversion efficacy. This randomized, double-blind, placebo-controlled, superiority clinical study is performed in patients with AF in order to evaluate the clinical efficacy of intravenous canrenone in sinus rhythm restoration.

**Methods:**

Eighty eligible patients with an episode of AF lasting less than 48 h are randomized in a 1:1 ratio to receive canrenone or placebo. Patients randomized to a treatment intervention are receiving canrenone intravenously at a dose of 200 mg within 2–3 min. Subjects assigned to a control group obtain the same volume of 0.9% saline within the same time. The primary endpoint includes return of sinus rhythm documented in the electrocardiogram within 2 h after drug or placebo administration. Other endpoints and safety outcomes analyses, due to expected lack of statistical power, are exploratory.

**Discussion:**

Current evidence supports renin–angiotensin–aldosterone system (RAAS) inhibition as an upstream therapy in AF management. Excess aldosterone secretion results in proarrhythmic effects. Among the RAAS inhibitors, only canrenone is administered intravenously. Canrenone additionally increases the plasma level of potassium, lowers blood pressure and reduces preload. It has been already used in primary and secondary hyperaldosteronism in the course of chronic liver dysfunction and in heart failure.

**Trial registration:**

ClinicalTrials.gov, NCT03536806. Registered on 25 May 2018.

## Background

Atrial fibrillation (AF) is the most frequent cardiac arrhythmia, being one of the major causes of emergency department visits and hospital admissions [1]. AF contributes to increased risk of thromboembolic complications and impaired quality of life. There are several approaches in AF management. One of them is an attempt to restore sinus rhythm (SR) with cardioversion, either pharmacological or electrical. According to ACC/AHA/HRS and ESC guidelines, application of class Ic (i.e. flecainide, propafenone) and class III (i.e. ibutilide, dofetilide, amiodarone, vernakalant) antiarrhythmic agents are recommended in pharmacological cardioversion of AF. Classes of recommendations depend on concomitant heart diseases [1, 2]. In general, the efficacy of antiarrhythmic agents is limited and their use may be associated with a potential risk of proarrhythmic and other adverse effects. Unlike symptomatic treatment, underlying conditions of arrhythmia are essential to be established and treated simultaneously. AF might be induced by electrolyte, neurohormonal disorders, increased blood pressure, atrial volume overload and inflammatory states. Therefore, there is a need to search for appropriate, effective and safe supplementing treatment which may increase the efficacy of antiarrhythmic drugs. It has been demonstrated that plasma aldosterone concentrations are increased in patients with AF episodes and are reduced after sinus rhythm restoration [3–5]. Current evidence supports renin–angiotensin–aldosterone (RAAS) inhibition with angiotensin-converting enzyme inhibitors (ACE-I), angiotensin receptor blockers (ARB) or, potentially, mineralocorticoid receptor antagonists (MRA) as an upstream therapy for AF management [6–9]. Among the RAAS-inhibiting agents, only canrenone (potassium canreonate) is administered intravenously. Canrenone is a specific antagonist of aldosterone - a competitive inhibitor of mineralocorticoid receptors. Spironolactone is a prodrug which is active after its conversion into canrenone and other metabolites. By inhibiting the effects of aldosterone, it increases aqueous and sodium diuresis. Canrenone decreases secretion of potassium and reabsorption of sodium and chloride in the distal renal tubule and thus it increases the plasma level of potassium, promotes diuresis, lowers blood pressure and reduces preload. Spironolactone is used in the treatment of primary or secondary hyperaldosteronism, heart failure, arterial hypertension, edemas and ascites due tocongestive heart failure and cirrhosis [10, 11]. We conduct a randomized, double-blind, placebo-controlled, superiority clinical study in patients with paroxysmal AF in order to evaluate the clinical efficacy of intravenous canrenone in sinus rhythm restoration.

## Methods

### Study objectives

The aim of the present pilot study is to evaluate the clinical efficacy of canrenone administered intravenously for sinus rhythm restoration among patients with paroxysmal AF. The rationale of carrying out the study was to find another safe mode of treatment which could facilitate conversion to sinus rhythm in a short period of time.

### Study design

This randomized, double-blind, placebo-controlled, superiority clinical study is actually carried out at the Institute of Cardiology, Warsaw, Poland. The study will include 80 patients presenting with an episode of AF lasting less than 48 h. All participants must sign an informed consent form. The study protocol conforms to the Standard Protocol Items: Recommendations for Interventional Trials (SPIRIT) 2013 statement (Additional file 1).

Approval for the study was obtained from the local ethics committee (IK-NP-0021-24/1553/16; February 9, 2016), Protocol no. 2.62/VII/16 version 1.0; February 8, 2016. Protocol modifications (e.g. changes to eligibility criteria, outcomes, analyses) will be reported to the relevant parties: the Office for Registration of Medicinal Products, Medical Devices and Biocidal Products, ClinicalTrials.gov and the local ethics committee.

### Eligibility

Patients with an AF episode lasting less than 48 h and documented in the ECG are potentially eligible for enrollment if the criteria presented in Table 1 are met.

**Table 1:** Inclusion and exclusion criteria

Inclusion criteria	
• Written informed consent for enrollment	
• Patients aged between 40 and 75 years	
• Atrial fibrillation episode lasting for less than 48 h, documented by the ECG	
• Potassium plasma level < 4.5 mmol/l	
• Systolic blood pressure > 120 mmHg	
• Stable cardiopulmonary status (according to attending physician’s assessment)	
• In the case of left ventricle injury suspicion or unclear medical history of cardiac insufficiency, enrollment will be possible after echocardiographic examination	
Exclusion criteria	
• No written informed consent for enrollment	
• Allergy to canrenone or spironolactone	
• Cardiac insufficiency or left ventricular ejection fraction < 40%	
• Systolic blood pressure ≤ 120 mmHg	
• History of canrenone treatment in the 30 days before enrollment	
• Average QRS rate > 160 per minute	
• Advanced hepatic (international normalized ratio > 1.5, aminotransferases > 3 times above normal) or renal failure (eGFR < 40 ml/min/1.73 m^2^)	
• History of acute coronary syndrome, coronary artery bypass grafting, transient ischemic attack or stroke within the previous 30 days	
• Pre-excitation syndrome (which has not been treated with accessory pathway ablation)	
• Atrial fibrillation due to valvular heart disease	
• Atrial fibrillation episode resulting in myocardial ischemia (chest pain, ischemic changes in the ECG)	
Antiarrhythmic agents	
• Rate control medications, such as β-blockers or calcium channel blockers (verapamil, diltiazem), are allowed in the preceding 2 h	
• Chronic antiarrhythmic therapy is not an exclusion criterion. Atrial fibrillation episode indicates for its low efficacy	

*ECG* electrocardiogram, *eGFR* estimated glomerular filtration rate, *QRS* complex in the ECG

### Study procedures

**Fig. 1:**
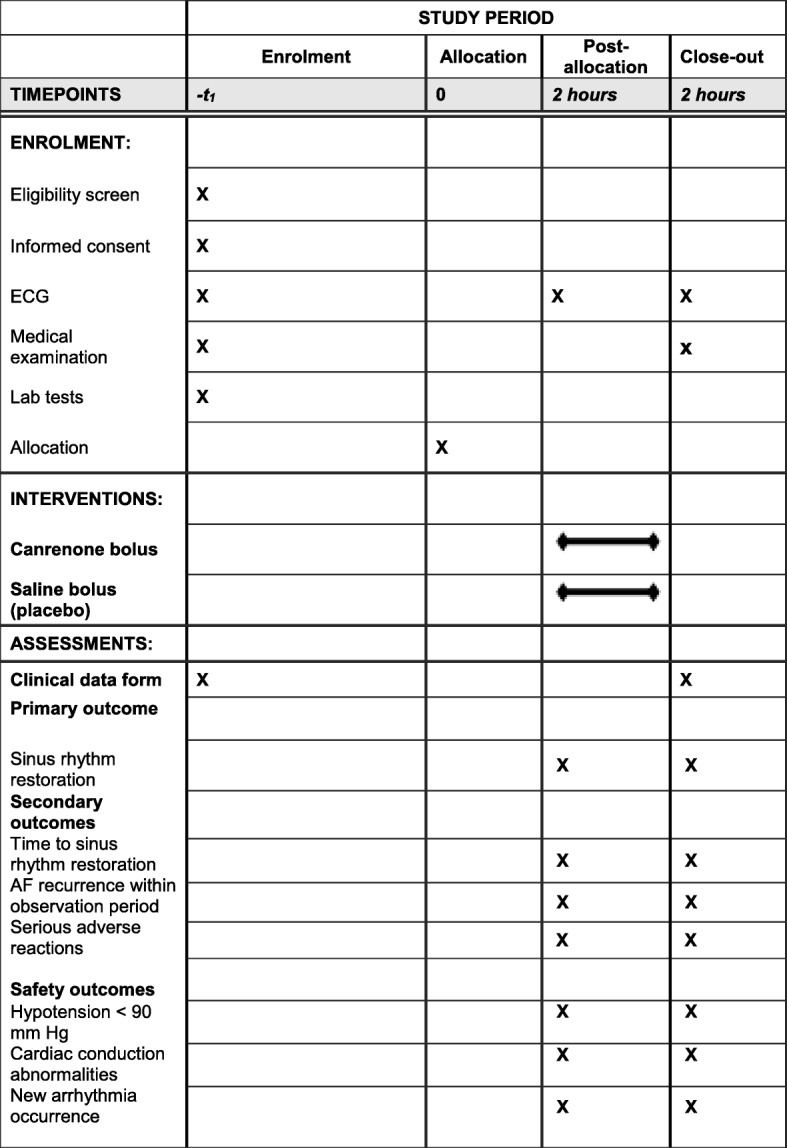
Schedule of enrolment, interventions and assessments. AF atrial fibrillation, ECG electrocardiogram

The schedule of enrollment, interventions and assessments is presented in Fig. 1.

Patients admitted to the Emergency department at the National Institute of Cardiology and diagnosis of a new onset atrial fibrillation are considered for participating in the study. Strategies for achieving adequate participant enrollment to reach the target sample size include graphic advertisements, periodical meetings with staff and repeated mailing actions.

All patients included in the trial, whether allocated to the study or control group, are managed in the same way according to protocols of pharmacological cardioversion. Such protocols are not specified by the trial, but they include standard components, comprising clinical evaluation, plasma electrolyte level assessment, baseline 12-lead ECG performance, continuous ECG monitoring, periodic noninvasive blood pressure (BP) control and an intravenous line.

Rate control medications, such as β-blockers or calcium channel blockers (diltiazem, verapamil), are allowed in the previous 2 h before study intervention. Chronic antiarrhythmic therapy is not an exclusion criterion—an AF episode indicates its low efficacy.

The assumptions of the study are explained to the patient and informed written consent is obtained. A case report form (CRF) is created for each patient. Investigators collect data regarding medical history, demographics, clinical presentation, previous and current treatment, and diagnostic test results. Both substances are prepared earlier in syringes by the study nurse unblinded to patient assignment. The study drug or placebo is administered intravenously in boluses by a nurse under the supervision of the enrolling physician, both blinded to patient assignment. In the case of serious adverse event occurrence, unblinding by the principal investigator is permissible, who will start the procedure for revealing a participant’s allocated intervention during the trial***.***

Each patient is observed within 2 h after the last bolus supply in order to determine efficacy and adverse reactions. The maximum concentration of canrenone is achieved within the first hour and decreases in two phases, with a mean half-life of 5–14 h, respectively [10].

**Fig. 2:**
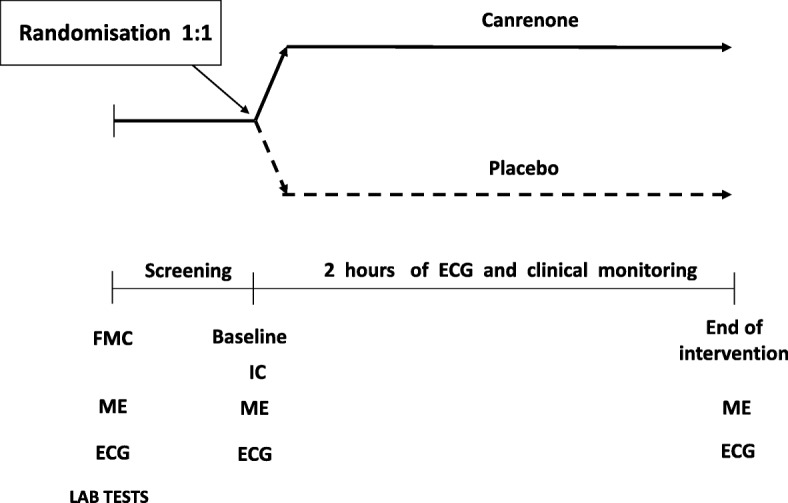
Study procedures and treatment assignment. ECG electrocardiogram, FMC first medical contact, IC informed consent, ME medical examination

When the 2-h observation period is over or when sinus rhythm returns, the ECG and blood pressure measurement valuation are performed. Further treatment of the patient will depend on their clinical condition and will follow appropriate clinical guidelines (Fig. 2) [1]. Enhanced monitoring for worsening of a subject’s condition and the use of standard rescue medications will be introduced in the case of need.

### Randomization

Randomization is provided by the independent statistician using SAS software (SAS Institute Inc., Cary, NC, USA). After eligible patients provide informed consent, they are assigned a specific identifier. Neither the patient nor the researcher knows which group the subject will be assigned to. Patients are randomized according to the implemented random allocation sequence using numbered sealed envelopes, which are opened after inclusion of the patient for the study in a 1:1 ratio to treatment with canrenone or placebo.

### Allocation concealment

Random allocation sequence is implemented using numbered sealed envelopes, which are opened after inclusion of the patient for the study.

### Interventions

#### Study group

Patients randomized to a study group will receive canrenone as a slow intravenous bolus within 2–3 min at a dose of 200 mg in a 10-ml ampule (Riemser Pharma). The drug administration is discontinued immediately after AF is terminated or in the case of any complex ventricular (ventricular tachycardia, fibrillation or flutter) or supraventricular (supraventricular tachycardia) arrhythmia as well as occurrence of any other serious adverse effects.

#### Control group

Patients assigned to a control group will receive 0.9% saline in a slow intravenous bolus in the same volume (i.e. 10 ml) within the same time (2–3 min). Saline administration will be stopped if AF is terminated or if any complex ventricular or supraventricular arrhythmia as well as any other serious adverse events occur.

#### Endpoints

The primary endpoint is sinus rhythm restoration documented in the ECG within 2 h after canrenone supply.

Secondary endpoints are the following:

- Time to sinus rhythm restoration
- AF recurrence within the observation period
- Serious adverse reactions, which refer to every event requiring admission to a hospital or extended observation
- Safety outcomes (exploratory analysis):
  - Hypotension < 90 mmHg
  - Cardiac conduction abnormalities
  - New arrhythmia occurrence

Data relating to the primary endpoint will be validated by a Clinical Committee. Data relating to safety will be monitored by a Safety Committee composed of three cardiologists with academic degrees experienced in conducting clinical trials. All adverse events are reported every month to the Safety Committee and the local ethics committee. These processes will be independent from investigators and the sponsor.

### Statistical methods

#### Sample size estimation

The sample size analysis is based on the *Z* test for fractions in two independent samples.

One of the purposes of this analysis is to compare fractions from two populations, with the hypothesis of their equality being tested. The *Z* test will be performed for two independent tests, with a type I error rate (α) of 0.01. The test is two-sided, so both positive and negative deviation from the hypothesis value can be considered significant. We assume that the population fractions in the groups are 0.50 and 0.05. In order to achieve 90.00% power, assuming that the sample numbers in the two groups are equal, a sample size of at least *N* = 27 from each group is required. For *N* = 27, the power in each group is 90.40%.

Assuming an optimistic scenario (no dropout interest and a sample of 80 people): Φ = Cramer’s *V* = 0.50396; relative benefit 10.0 [2.50–39.98].

The purpose of the effect size is to avoid a statistically significant result resulting only from a huge sample size, not from clinical effectiveness. Such a situation would not occur in the presented research. In conclusion, to show superiority of canrenone over placebo, a sample size of 80 patients was calculated based on the following assumptions: a two-tailed test, a type I error of 0.01, a power of 90%, efficacy of placebo of 5% (evaluated according to three trials testing vernakalant), efficacy of canrenone of 50% and a 20% dropout rate to fulfill the criteria of intention-to-treat analysis [12–14]. Due to a presumed low frequency of events, the secondary and safety endpoints will be evaluated in an exploratory manner.

#### Data management

During the study, the investigator regularly enters information into case report forms (CRFs). Database management and quality control of this study are the responsibility of a biostatistician. Structured data elements from the CRFs are entered into the database and reviews using double data entry for verification. Electronic data are kept in a duplex database—one available in the hospital department and the other available at one of the Safety Committee member’s department. Information entered into the database is systematically checked and obvious errors are corrected. Omissions or questions are returned to the investigator for resolutions. Paper data related to our study are preserved in the 2nd Department of Coronary Artery Disease.

### Statistical analysis

Comparison of categorical variables (sinus rhythm restoration, AF recurrence, serious adverse reactions, safety outcome) between groups will be performed using the χ^2^ test of independence with Yate’s correction, or with Fisher’s exact test in cases of minimum expected count less than 5, and these will be expressed as number and percentage.

After initial assessment of the quantitative variables’ distributions (time to sinus rhythm restoration, SBP, DBP, HR), measures of location and dispersion will be calculated—as arithmetic mean and standard deviation for normally distributed variables, or as median and quartile range for variables for which the Shapiro–Wilk test null hypothesis has been rejected. For comparison of continuous variables between the two groups, the unpaired Student’s *t* test (or Cochran’s *C* test in the case of heteroscedasticity of variance) will be used, or for skewed data, the nonparametric Mann–Whitney *U* test. Differences for matched samples (within groups) will be evaluated with McNemar’s test, paired Student’s *t* test or Wilcoxon’s rank sum test.

All probability values will be calculated from two-sided tests, and *P* < 0.05 will be considered statistically significant. All statistical analysis will be performed using SAS 9.42 software (SAS Institute Inc.). After inclusion of 20 patients, effectivity and safety analyses will be performed by the statistician, Safety Committee and principal investigator, who will have access to interim results and will make the final decision to continue or terminate the trial. Hypotheses will be tested according to the significance level set to α ≤ 0.05 with 2-sided tests. Statistical analysis will be conducted using SAS 9.2 software.

## Discussion

There is a growing body of evidence from experimental and clinical studies that management of AF should include upstream therapy. Studies’ results confirm that drugs affecting the RAAS have a positive impact on presumable substrates of arrhythmia and its comorbidities. Increased neurohormonal activity (i.e. boosted levels of angiotensin II, aldosterone, catecholamines), a common mechanism underlying cardiovascular pathologies, leads to structural and electrical atrial and ventricle remodeling. Thus, careful follow-up and supervision, including adequate hydration and electrolyte balance maintenance, treatment of hypertension, heart failure, coronary artery disease and other comorbidities, may possibly correct the background of arrhythmia. Due to limited efficacy and adverse effects of class I, III and IV antiarrhythmic agents (except for β-blockers), an additional therapeutic approach could be administration of mineralocorticoid receptor antagonists (MRA). It is important to start MRA in the early stages of AF also to prevent fibrosis and more complex forms of arrhythmia [7, 8].

In patients suffering from recurrent AF episodes treatment with ACE-I or ARB (class of recommendation: IIb/B) and/or aldosterone antagonists may byconsidered to reduce arrhythmia burden and to prevent heart failure. These drugs can be administered in order to protect patients from arrhythmia recurrence in structurally normal hearts even if recommended in other medical indications (hypertension, heart failure) (class of recommendation: IIa/B). Proper extensive use of upstream therapy is estimated to reduce global burdens and consequences of AF for healthcare systems even by 30% [1, 2, 7, 9]. Additionally, the issue of improvement of cardioversion efficacy is of importance.

Currently recommended medications (i.e. propafenone, flecainide, amiodarone, vernakalant) may cause adverse reactions and have significant contraindications [1, 2]. Therefore, we assume that evaluation of intravenous canrenone efficacy (a specific aldosterone antagonist and a major metabolite of spironolactone) in sinus rhythm restoration in patients with AF episode may be beneficial. The effect of canrenone starts in 2 h. It has been demonstrated that plasma aldosterone is increased in patients with AF episodes, whereas it lowers after successful cardioversion [3–5]. Excess of aldosterone may result in proarrhythmic effects. Canrenone, a major metabolite of spironolactone, suppresses aldosterone directly. It maintains potassium balance and acts as a diuretic (hence reducing preload and atrial volume overload). Canrenone also inhibits fibrosis. These are mechanisms of AF underlying pathology. There is evidence showing that ramipril with oral canrenone is superior to ramipril with hydrochlorothiazide in the prevention of AF episode recurrence [15, 16].

## Trial status

Recruitment has started since January 1, 2019 according to Protocol no. 2.62/VII/16 version 1.0; February 8, 2016. At the time of manuscript submission,the expected duration of the study, including enrollment and statistical analysis, should be 3 years. The approximate date of planned recruitment completion is December 31, 2021.

## Supplementary information

**Additional file 1.** SPIRIT 2013 Checklist: Recommended items to address in a clinical trial protocol and related documents.

## Data Availability

The study investigators have full access to study datasets. The datasets used and analyzed during the study are available from the corresponding author on reasonable request; however, any information shared will be blinded to any identifying participant information. Trial results will be communicated to healthcare professionals and other relevant groups via publications, reporting in results databases and presenting the data during the medical congresses and conferences.

## Abbreviations

ACE-I: Angiotensin-converting enzyme inhibitors
AF: Atrial fibrillation
ARB: Angiotensin receptor blockers
BP: Blood pressure
CRF: Case report form
ECG: Electrocardiogram
MRA: Mineralocorticoid receptor antagonists
RAAS: Renin–angiotensin–aldosterone system
SR: Sinus rhythm
